# Expanding the phenotype of de novo *SLC25A4*-linked mitochondrial disease to include mild myopathy

**DOI:** 10.1212/NXG.0000000000000256

**Published:** 2018-07-20

**Authors:** Martin S. King, Kyle Thompson, Sila Hopton, Langping He, Edmund R.S. Kunji, Robert W. Taylor, Xilma R. Ortiz-Gonzalez

**Affiliations:** From the Medical Research Council Mitochondrial Biology Unit (M.S.K., E.R.S.K.), University of Cambridge, Wellcome Trust/MRC Building, Cambridge Biomedical Campus, UK; Wellcome Centre for Mitochondrial Research (K.T., S.H., L.H., R.W.D.), Institute of Neuroscience, Newcastle University, UK; and Department of Neurology (X.R.O.), Perelman School of Medicine, Division of Neurology and Center for Mitochondrial and Epigenomic Medicine, The Children's Hospital of Philadelphia, University of Pennsylvania.

## Abstract

**Objective:**

To determine the disease relevance of a novel de novo dominant variant in the *SLC25A4* gene, encoding the muscle mitochondrial adenosine diphosphate (ADP)/adenosine triphosphate (ATP) carrier, identified in a child presenting with a previously unreported phenotype of mild childhood-onset myopathy.

**Methods:**

Immunohistochemical and western blot analysis of the patient's muscle tissue were used to assay for the evidence of mitochondrial myopathy and for complex I–V protein levels. To determine the effect of a putative pathogenic p.Lys33Gln variant on ADP/ATP transport, the mutant protein was expressed in *Lactococcus lactis* and its transport activity was assessed with fused membrane vesicles.

**Results:**

Our data demonstrate that the heterozygous c.97A>T (p.Lys33Gln) *SLC25A4* variant is associated with classic muscle biopsy findings of mitochondrial myopathy (cytochrome c oxidase [COX]-deficient and ragged blue fibers), significantly impaired ADP/ATP transport in *Lactococcus lactis* and decreased complex I, III, and IV protein levels in patient's skeletal muscle. Nonetheless, the expression levels of the total ADP/ATP carrier (AAC) content in the muscle biopsy was largely unaffected.

**Conclusions:**

This report further expands the clinical phenotype of de novo dominant *SLC25A4* mutations to a childhood-onset, mild skeletal myopathy, without evidence of previously reported clinical features associated with *SLC25A4*-associated disease, such as cardiomyopathy, encephalopathy or ophthalmoplegia. The most likely reason for the milder disease phenotype is that the overall AAC expression levels were not affected, meaning that expression of the wild-type allele and other isoforms may in part have compensated for the impaired mutant variant.

*SLC25A4* (*ANT1* and *AAC1*) gene mutations cause an intriguing spectrum of human disease, with dominant mutations first reported in adults with progressive external ophthalmoplegia (PEO),^1^ whereas recessive loss-of-function mutations cause cardiomyopathy and skeletal myopathy.^2,3^ Furthermore, *SLC25A4* de novo dominant mutations can present in neonates with lactic acidosis, severe hypotonia, and respiratory failure.^4^ Here, we report a patient who presented at age 2 years with mild weakness and hypotonia and was found to have a novel de novo heterozygous *SLC25A4* variant (c.97A>T;p.Lys33Gln).

The *SLC25A4* gene encodes the mitochondrial AAC1, which imports ADP into the mitochondrion and exports ATP.^5^ Humans have 4 AAC isoforms, with AAC1 being specific to the heart, skeletal muscle, and brain.^6^ Previous functional studies correlating the rate of ADP/ATP exchange with phenotype severity have only partially solved the puzzle. In dominant-acting mutations, the rate of transport does seem to correlate with clinical severity, with mutations associated with PEO having higher residual ADP/ATP transport rates compared with de novo mutations associated with severe neonatal disease.^4^ However, loss-of-function recessive mutations present later than de novo cases with a predominant cardiac phenotype, despite in vitro functional studies showing essentially no measurable ADP/ATP exchange^4^

Here, we report that the de novo dominant variant p.Lys33Gln in *SLC25A4* is clinically associated with mild myopathy despite significant mitochondrial pathology in muscle biopsy and functional data showing abolished ADP/ATP exchange in vitro. This case expands the known phenotype of *SLC25A4* disease to include childhood-onset mild skeletal myopathy without evidence of cardiac, brain, or extraocular muscle involvement.

## Methods

### Case presentation

A 2-year old girl presented to the neurology clinic at the Children's Hospital of Philadelphia (CHOP) for hypotonia and mild gross motor delays. Neurologic examination at presentation was only remarkable for hypotonia and a 1-handed Gower maneuver, suggestive of mild weakness. Family history was unremarkable. Laboratory workup found elevated creatine kinase (CK) (616 U/L, normal 60–305) and lactic acidosis (3.76 mM, normal 0.8–2.0) levels; therefore, muscle biopsy and subsequent genetic testing were pursued. Clinical testing for nuclear mitochondrial disease genes and full mitochondrial DNA (mtDNA) sequencing (in blood and muscle) were obtained via the next-generation sequencing panel.

### Standard protocol approvals, registrations, and patient consents

Informed consent was obtained from the child's parents to enroll in a human subject's research protocol approved by the Institutional Review Board at CHOP.

### Human muscle immunohistochemistry and analysis

Standard histologic and histochemical analyses were performed on 10-µm transversely oriented muscle cryosections. Quadruple immunofluorescence analysis of NDUFB8 (complex I) and COXI (complex IV)^7^ and western blot analysis^3^ were performed as previously reported. mtDNA copy number assessment in muscle was undertaken as described.^8^

### Functional studies of p.Lys33Gln variant in *Lactococcus lactis*

The *SLC25A4* gene was cloned into the *L. lactis* expression vector pNZ8048 by established procedures,^9^ and the p.Lys33Gln variant was introduced and confirmed by sequencing. Growth of *L. lactis*, membrane isolation, vesicle preparation, transport assays, and western blot analysis were performed as reported.^4,9^

## Results

### Patient results

**Figure 1:**
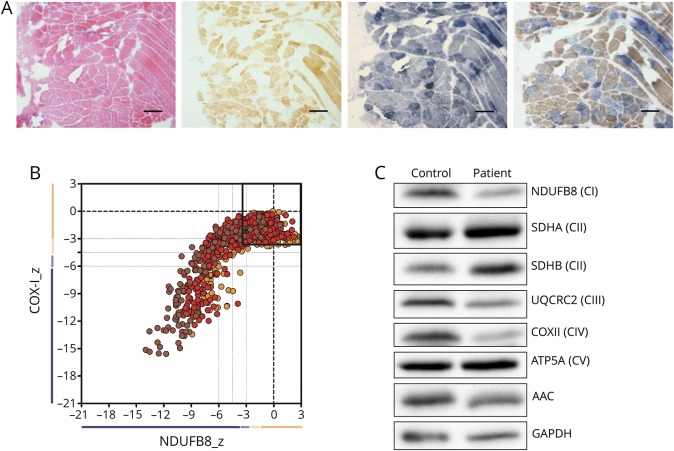
The de novo p.Lys33Gln mutation leads to OXPHOS defect in muscle (A) Histopathologic analysis of patient skeletal muscle sections showing hematoxylin and eosin (H&E) staining (far left), COX histochemistry (middle left), SDH histochemistry (middle right), and sequential COX-SDH histochemistry (far right). Scale bar = 100 µm. (B) Respiratory chain profile following quadruple oxidative phosphorylation immunofluorescence analysis of cryosectioned muscle from the index case, confirming the presence of fibers lacking complex I (NDUFB8) and complex IV (COXI) protein. Each dot represents the measurement from an individual muscle fiber, color coded according to its mitochondrial mass (blue-low, normal-beige, high-orange, and very high–red). Gray dashed lines indicate SD limits for the classification of fibers. Lines next to x- and y-axes represent the levels (SDs from the average of control fibers after normalization to porin/VDAC1 levels; _z= Z-score, see Methods section of Rocha et al. 2015 for full description of statistics^7^) of NDUFB8 and COX1, respectively (beige = normal [>−3], light beige = intermediate positive [−3 to−4.5], light purple = intermediate negative [−4.5 to −6], and purple = deficient [<−6]). Bold dotted lines indicate the mean expression level observed in respiratory normal fibers. (C) Western blot analysis of AAC and OXPHOS complex subunits on control and patient skeletal muscle samples. AAC = ADP/ATP carrier; ADP = adenosine diphosphate; ATP = adenosine triphosphate; COX = cytochrome c oxidase; GADPH = Glyceraldehyde 3-phosphate dehydrogenase; OXPHOS = oxidative phosphorylation; SDH = succinate dehydrogenase.

Histopathologic assessment of skeletal muscle from the patient demonstrated a mosaic pattern of cytochrome c oxidase (COX) deficiency (figure 1A). Quadruple immunofluorescence analysis confirmed a mitochondrial defect involving both complexes I and IV with some fibers exhibiting normal protein expression (figure 1B). MtDNA copy number was decreased to approximately 40% in patient muscle compared with age-matched controls. Western blot analysis showed a slight decrease in AAC protein levels, associated with more markedly decreased steady-state protein levels of components of respiratory complexes (CI, CIII, and CIV) in patient skeletal muscle (figure 1C).

Genetic testing revealed a heterozygous *SLC25A4* variant (c.97A>C, p.Lys33Gln) that was confirmed to have arisen de novo following parental testing. Serial cardiac evaluations including ECG, echocardiogram, and Holter monitoring were unremarkable from diagnosis at age 2 years to current age of 8 years. Neurologic evaluations remain stable, only remarkable for mild proximal weakness, hyperCKemia, and lactic acidosis, with normal extraocular movements and no cognitive abnormalities.

### Assessment of *SLC25A4* p.Lys33Gln variant function

**Figure 2:**
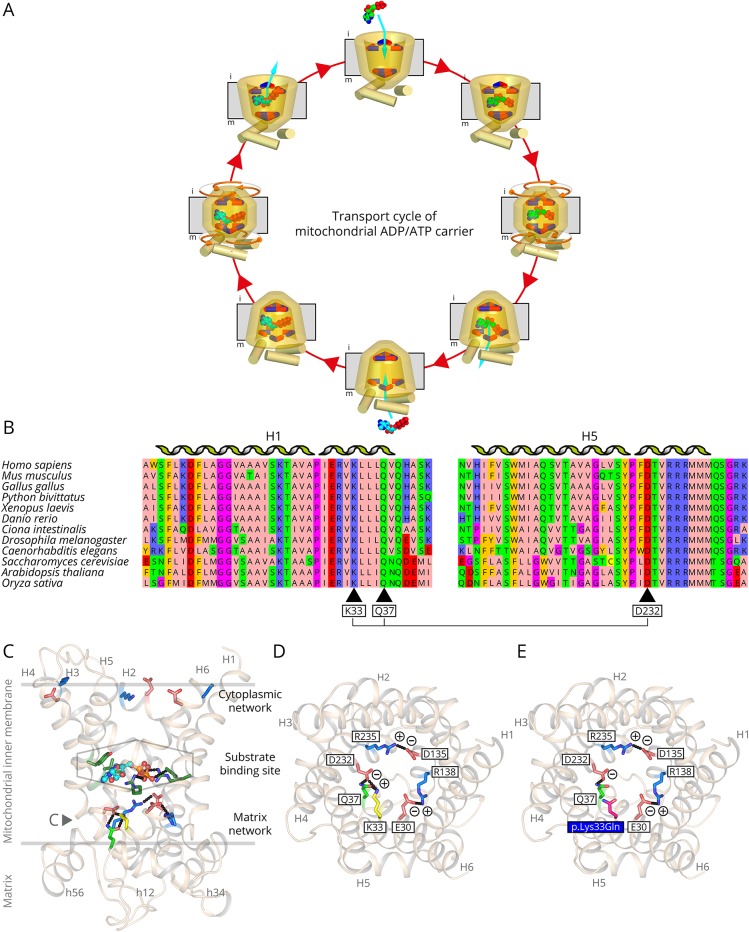
The p.Lys33Gln mutation eliminates a conserved salt bridge interaction of the matrix network (A) Transport cycle of the mitochondrial AAC SLC25A4. The disruption and formation of salt bridges between positively (blue) and negatively (red) charged residues of the cytoplasmic and matrix networks, top and bottom respectively, change the access of the substrates to the central substrate binding site, indicated by a hexagon, from the intermembrane space (i) and matrix (m) side of the membrane. The imported ADP is shown in green sphere representation and the exported ATP in cyan. (B) Amino acid sequence alignment of AAC1 sequences of fungi, plants, and metazoan, showing that Lys33, Gln37, and Asp232 are highly conserved amino acid residues. (C) Lateral view of the human ADP/ATP carrier from the membrane, showing the residues of the matrix and cytoplasmic networks (blue and red sticks) and substrate binding site (green sticks, hexagon). ADP (light blue ball and stick) and the glutamine brace (light green stick) are also shown. Residue Lys33 that is mutated is shown in yellow. (D) Cytoplasmic view of the carrier showing only the residues of the matrix salt bridge network of SLC25A4 (blue and red sticks). (E) As (D), except for the p.Lys33Gln mutation, which is shown in magenta. The ionic interactions (black dash lines) are indicated with plus and minus signs. The model of human SLC25A4 was generated in SwissModel, using the structure of the closely related bovine mitochondrial AAC as template (PDB file: 1OKC). Adapted from Figure 3 in reference 4. AAC = ADP/ATP carrier; ADP = adenosine diphosphate; ATP = adenosine triphosphate.

The transport mechanism of SLC25A4 involves the disruption and formation of the matrix and cytoplasmic salt bridge network in an alternative way (figure 2A).^5^ Residue Lys33 in SLC25A4, which is conserved among AAC from fungi, plants, and metazoans (figure 2B), forms a salt bridge with the conserved Asp232 in the matrix network (figure 2, C and D). The p.Lys33Gln mutation would eliminate this interaction, as glutamine is a neutral amino acid residue and too short to form a hydrogen bond (figure 2E). Below the salt bridge is Gln37 that forms a highly conserved glutamine brace^10^ (figure 2, B–D), which would also be disrupted by the mutation (figure 2E).

**Figure 3:**
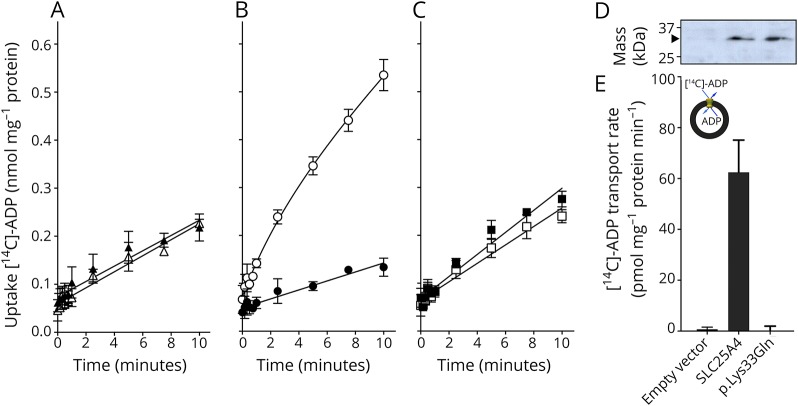
Transport activity of human SLC25A4 and SLC25A4 p.Lys33Gln Transport of [^14^C]-labeled ADP into vesicles of *L. lactis* membranes expressing (A) an empty vector control, (B) SLC25A4, or (C) SLC25A4 p.Lys33Gln in the presence (black symbols) or absence (white symbols) of 20 µM carboxyatractyloside. Transport was initiated by the addition of 5 µM [^14^C]-ADP and was terminated by filtration and washing at the indicated time intervals. The data are represented by the average and SD of 4 assays. (D) Expression levels of wild type and p.Lys33Gln determined by western blot analysis. (E) Transport rate of the empty vector control, human SLC25A4, and p.Lys33Gln, corrected for background binding. AAC = ADP/ATP carrier; ADP = adenosine diphosphate; ATP = adenosine triphosphate.

We introduced the p.Lys33Gln mutation into the human *SLC25A4* sequence and expressed it *L. lactis* membranes. The uptake of radio-labeled ADP in exchange for loaded ADP was measured for an empty vector control (figure 3A), wild-type SLC25A4 (figure 3B), and p.Lys33Gln, using the specific inhibitor carboxyatractyloside as control (figure 3C). The mutant protein is expressed to approximately the same levels as wild-type in lactococcal membranes, suggesting that the mutation does not affect biogenesis, protein folding, or targeting to the membrane in this expression system (figure 3D). The p.Lys33Gln mutant was not able to transport ADP (figure 3E).

## Discussion

Previously, we have functionally characterized the effect of 9 pathogenic variants in human *SLC25A4* using *L. lactis*, determining residual transport activity compared with wild-type protein.^4^ Broadly speaking, residual transport activities of the mutants segregate with the associated clinical phenotype; mutations associated with adult-onset dominant PEO display higher residual transport activities (24%–56%) than mutations associated with recessive disease, which were effectively nonfunctional.^4^ There was also a correlation between the severity of the clinical phenotype and residual transport activity of previously documented dominant mutations, with mutations associated with inherited PEO showing higher activity than mutations associated with de novo severe neonatal presentation.^3^

It remains unclear exactly why the presently documented case with the de novo dominant c.97A>T(p.Lys33Gln) *SLC25A4* variant presents with a much milder clinical phenotype than the other de novo cases^3^ despite the p.Lys33Gln mutant protein revealing essentially null ADP/ATP transport activity (figure 3E). This case further supports that residual ADP/ATP transport activity of mutant AAC1 is only part of the puzzle. In vivo, there are 3 other AAC isoforms with varying expression levels in different tissues. Also, since the de novo variants are heterozygous, the relative expression ratios of wild-type vs mutant AAC1, as well as other isoforms, will have an effect on the overall transport capacity. Their relative expression levels of carriers cannot be assessed from messenger RNA levels, as there are many poorly characterized steps involved in their biogenesis and turnover, and the available antibodies are unable to distinguish between the various isoforms.

Despite these issues, there is a clear correlation between the severity of OXPHOS dysfunction in skeletal muscle and the clinical phenotype between the current and previously reported de novo mutations. Patients with p.Arg80His or p.Arg235Gly mutations, showing severe neonatal presentations, had markedly decreased levels of total AAC (<30%),^3^ whereas there is only a slight decrease in the current patient (figure 1C). Similarly, the mosaic pattern of OXPHOS deficiency (figure 1, A and B) is not seen in previously documented de novo cases, where steady state protein levels of various OXPHOS components were completely undetectable.^3^ These data suggest that the clinical severity of de novo dominant mutations in *SLC25A4* could be explained by the relative expression of wild-type and mutant alleles and the expression of other AAC isoforms, rather than the transport activity of the mutant variant alone.

This report expands the clinical phenotype for *SLC25A4*-associated mitochondrial disease, with the mildest childhood-onset presentation to date.

## Glossary:

AAC: ADP/ATP carrier
ADP: adenosine diphosphate
ATP: adenosine triphosphate
COX: cytochrome c oxidase
CHOP: Children's Hospital of Philadelphia
CK: creatine kinase
mtDNA: mitochondrial DNA
OXPHOS: oxidative phosphorylation
PEO: progressive external ophthalmoplegia

## References

[1] Kaukonen J, Juselius JK, Tiranti V, et al Role of adenine nucleotide translocator 1 in mtDNA maintenance. Science 2000;289:782–785. 10926541 10.1126/science.289.5480.782

[2] Palmieri L, Alberio S, Pisano I, et al Complete loss-of-function of the heart/muscle-specific adenine nucleotide translocator is associated with mitochondrial myopathy and cardiomyopathy. Hum Mol Genet 2005;14:3079–3088. 16155110 10.1093/hmg/ddi341

[3] Strauss KA, Dubiner L, Simon M, et al Severity of cardiomyopathy associated with adenine nucleotide translocator-1 deficiency correlates with mtDNA haplogroup. Proc Natl Acad Sci USA 2013;110:3453–3458. 23401503 10.1073/pnas.1300690110 PMC3587196

[4] Thompson K, Majd H, Dallabona C, et al Recurrent de novo dominant mutations in SLC25A4 cause severe early-onset mitochondrial disease and loss of mitochondrial DNA copy number. Am J Hum Genet 2016;99:860–876. 27693233 10.1016/j.ajhg.2016.08.014 PMC5065686

[5] Kunji ER, Aleksandrova A, King MS, et al The transport mechanism of the mitochondrial ADP/ATP carrier. Biochim Biophys Acta 2016;1863:2379–2393. 27001633 10.1016/j.bbamcr.2016.03.015

[6] Dolce V, Scarcia P, Iacopetta D, Palmieri F A fourth ADP/ATP carrier isoform in man: identification, bacterial expression, functional characterization and tissue distribution. FEBS Lett 2005;579:633–637. 15670820 10.1016/j.febslet.2004.12.034

[7] Rocha MC, Grady JP, Grunewald A, et al A novel immunofluorescent assay to investigate oxidative phosphorylation deficiency in mitochondrial myopathy: understanding mechanisms and improving diagnosis. Sci Rep 2015;5:15037. 26469001 10.1038/srep15037 PMC4606788

[8] Blakely E, He L, Gardner JL, et al Novel mutations in the TK2 gene associated with fatal mitochondrial DNA depletion myopathy. Neuromuscul Disord 2008;18:557–560. 18508266 10.1016/j.nmd.2008.04.014

[9] King MS, Boes C, Kunji ER Membrane protein expression in Lactococcus lactis. Methods Enzymol 2015;556:77–97. 25857778 10.1016/bs.mie.2014.12.009

[10] Ruprecht JJ, Hellawell AM, Harding M, Crichton PG, Mccoy AJ, Kunji ERS Structures of yeast mitochondrial ADP/ATP carriers support a domain-based alternating-access transport mechanism. Proc Natl Acad Sci USA 2014;111:E426–E434. 24474793 10.1073/pnas.1320692111 PMC3910652

